# Supplementary material for: Assessing the causal association of glycine with risk of cardio-metabolic diseases
Source: Nat Commun. 2019 Mar 5;10:1060. doi: 10.1038/s41467-019-08936-1 (PMC6400990; doi:10.1038/s41467-019-08936-1)
Supplement: Supplementary file 1 — Supplementary Information [file 41467_2019_8936_MOESM1_ESM.pdf]

Assessing the causal association of glycine with risk of cardio-metabolic diseases

Laura B. L. Wittemans *et al.*

Supplementary Information

Supplementary Figures

Supplementary Figure 1: Forest plots of study-specific effect sizes of the 27 significant loci on glycine in the Fenland, EPIC-Norfolk and INTERVAL studies. **p3-5**

Supplementary Figure 2: Sex-combined and sex-specific per-allele effect sizes of rs715 (*CPS1*) on metabolite levels. **p6**

Supplementary Figure 3: Dosage plots of effect sizes of glycine variants on SD of glycine levels versus the log odds for CHD in women. **p7**

Supplementary Figure 4: Dosage plots of effect sizes of glycine variants on SD of glycine levels versus the log odds for CHD in men. **p8**

Supplementary Figure 5: Forest plot of genetically predicted odds ratios on stroke and stroke sub-types per SD of genetically predicted glycine levels, based on 4 genetic scores for glycine. **p9**

Supplementary Figure 6: Effect sizes of rs715 on systolic and diastolic blood pressure, by sex and for sexes combined. **p10**

Supplementary Figure 7: Forest plots showing the effect sizes of genetically predicted glycine levels by the 4 genetic scores on 19 risk factors of CHD. **p11**

Supplementary Figure 8: Dosage plots of the effect sizes of genetic variants for glycine on standard deviations of glycine levels versus the log odds for type 2 diabetes for sex-combined analyses. **p12**

Supplementary Figure 9: Dosage plots of the effect sizes of genetic variants for glycine on standard deviations of glycine levels versus the log odds for type 2 diabetes for women-only analyses. **p13**

Supplementary Figure 10: Dosage plots of the effect sizes of genetic variants for glycine on standard deviations of glycine levels versus the log odds for type 2 diabetes for men-only analyses. **p14**

Supplementary Tables

Supplementary Table 1: Overview of samples, phenotype measurement and transformation, genotyping and imputation for the 5 studies included in the p-value and sample size-based meta-analysis of GWAS for glycine levels. **p15**

Supplementary Table 2: Results of Mendelian randomisation analyses of glycine to CHD, sex-specific and combined, and using 4 different methods and 4 different genetic scores for glycine. **p16-17**

Supplementary Table 3: Results of Mendelian randomisation analyses of glycine to stroke and stroke sub-types, using 4 different methods and 4 different genetic scores for glycine. **p18-19**

Supplementary Table 4: Results of Cox proportional hazards models for the association of glycine levels with CHD, myocardial infarction and stroke (including stroke subtypes). **p20**

Supplementary Table 5: results of inverse variance-weighted Mendelian randomisation analyses for the assessment of the effect of glycine levels on 19 CHD risk factors. **p21-22**

Supplementary Table 6: Results of Mendelian randomisation analyses of glycine to T2D, sex-specific and combined, and using 4 different methods and 4 different genetic scores for glycine. **p23-24**

Supplementary Table 7: Reverse Mendelian randomisation analyses to assess the causality of T2D risk factors on glycine levels. **p25**

Supplementary Table 8: Results of Mendelian randomisation analyses of glycine to 3 site-specific cancers. **p26-27**

Supplementary Note 1: Acknowledgements. p28-30

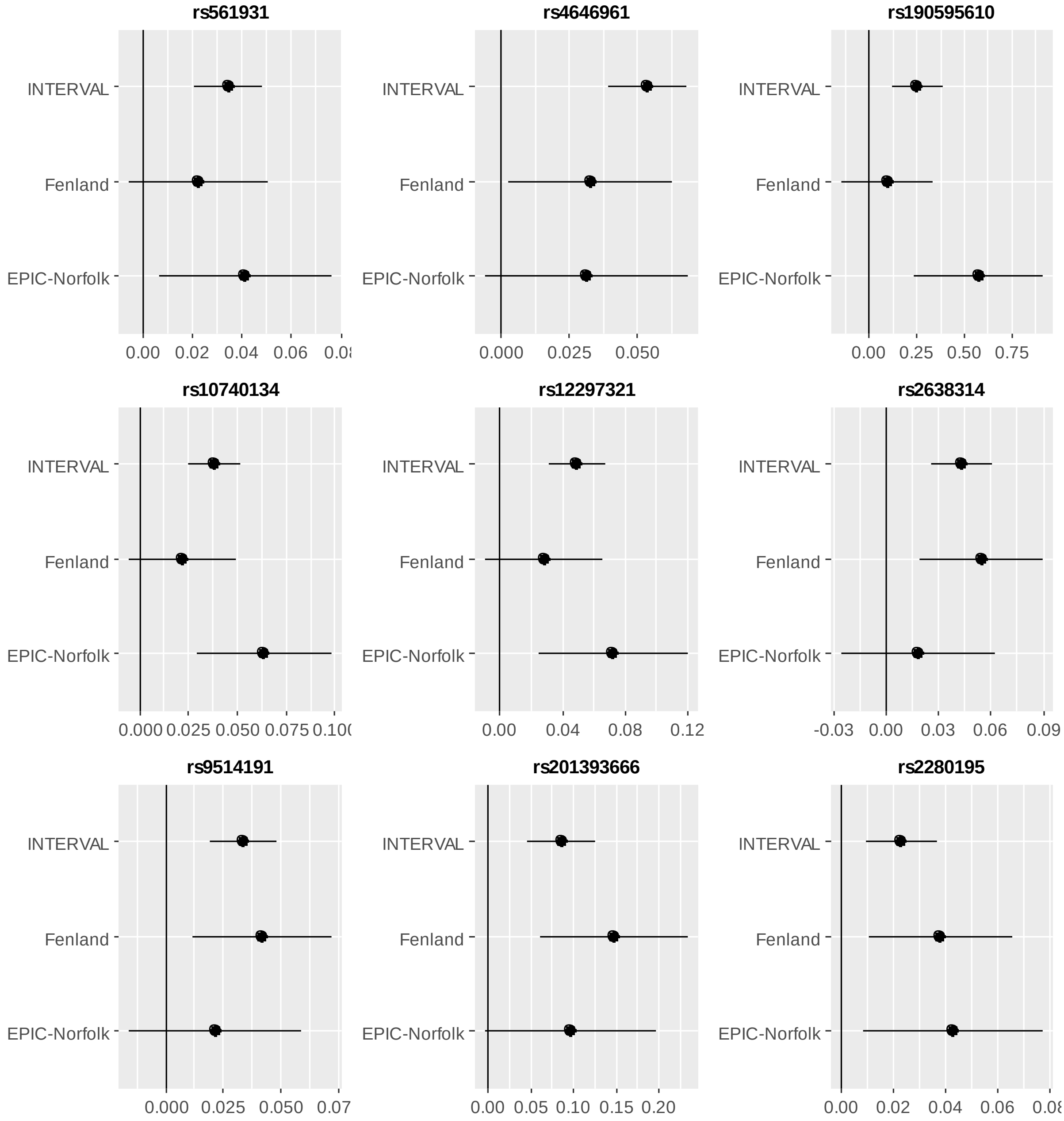

rs561931
rs4646961
rs190595610
rs10740134
rs12297321
rs2638314
rs9514191
rs201393666
rs2280195
INTERVAL
Fenland
EPIC-Norfolk

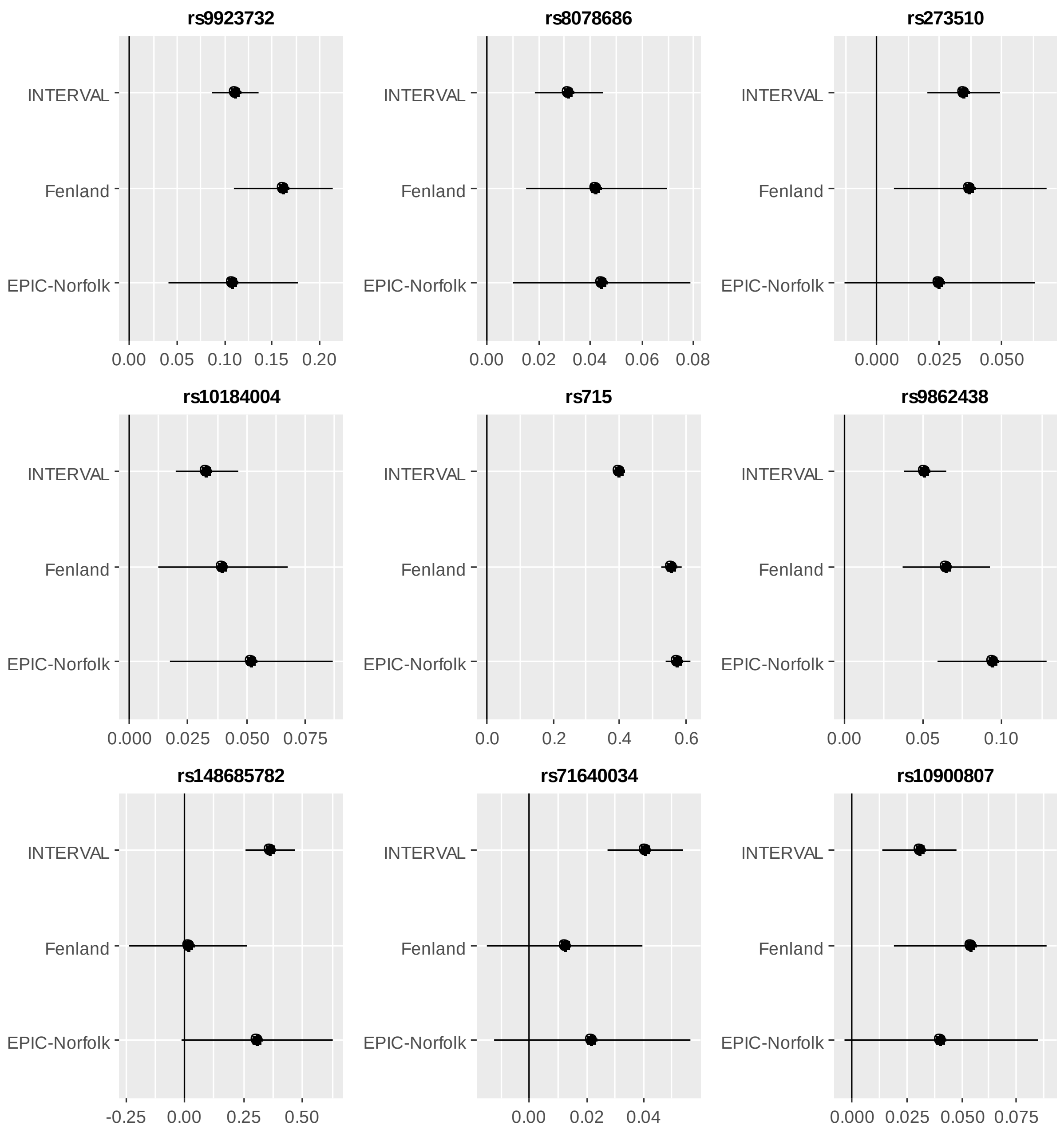
rs9923732
INTERVAL
Fenland
EPIC-Norfolk
0.00 0.05 0.10 0.15 0.20
rs8078686
INTERVAL
Fenland
EPIC-Norfolk
0.00 0.02 0.04 0.06 0.08
rs273510
INTERVAL
Fenland
EPIC-Norfolk
0.000 0.025 0.050
rs10184004
INTERVAL
Fenland
EPIC-Norfolk
0.000 0.025 0.050 0.075
rs715
INTERVAL
Fenland
EPIC-Norfolk
0.0 0.2 0.4 0.6
rs9862438
INTERVAL
Fenland
EPIC-Norfolk
0.00 0.05 0.10
rs148685782
INTERVAL
Fenland
EPIC-Norfolk
-0.25 0.00 0.25 0.50
rs71640034
INTERVAL
Fenland
EPIC-Norfolk
0.00 0.02 0.04
rs10900807
INTERVAL
Fenland
EPIC-Norfolk
0.000 0.025 0.050 0.075

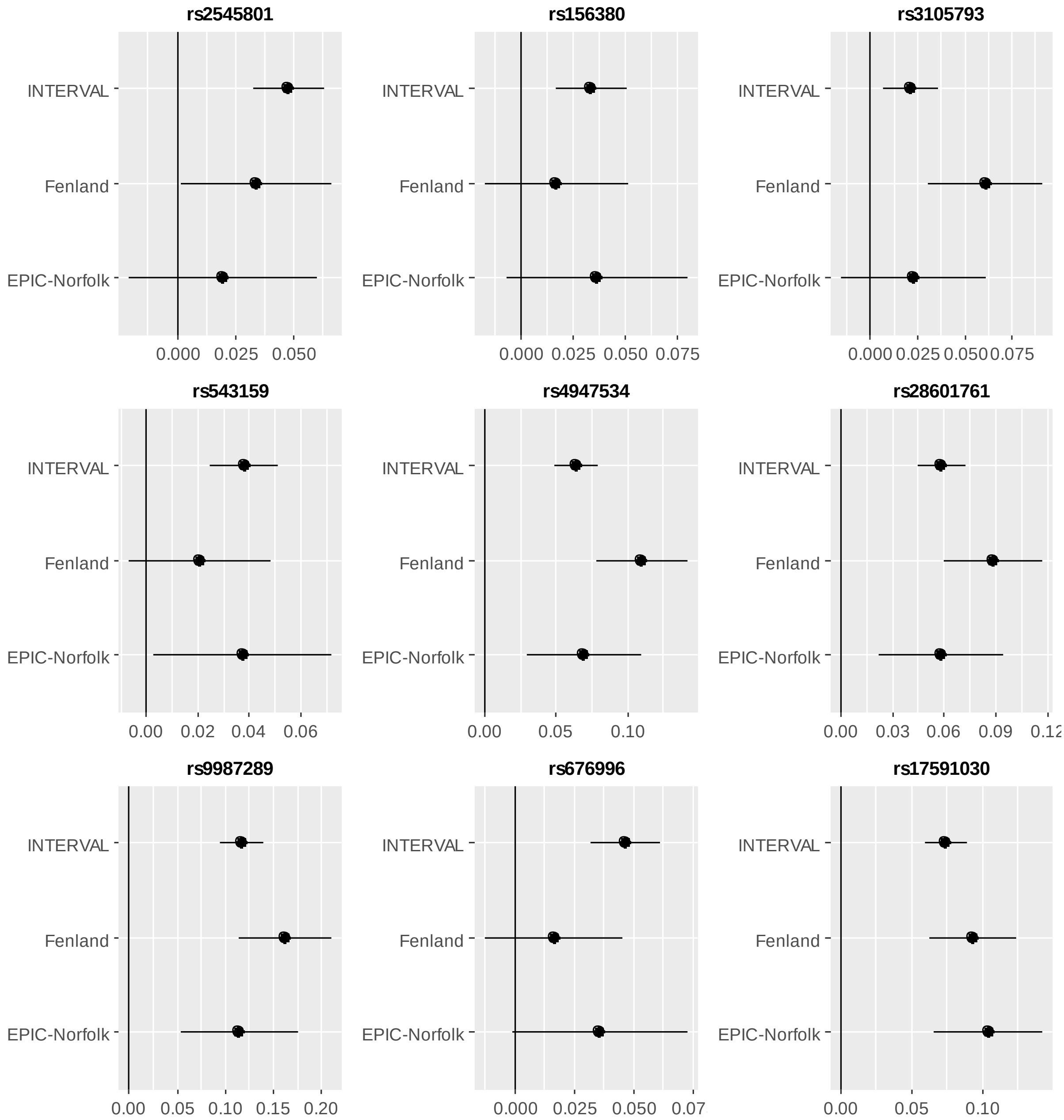

Supplementary Figure 1: Forest plots of study-specific effect sizes of the 27 significant loci on glycine in the Fenland, EPIC-Norfolk and INTERVAL studies. The x axes represent the per-allele effect size on standard deviations of glycine levels.

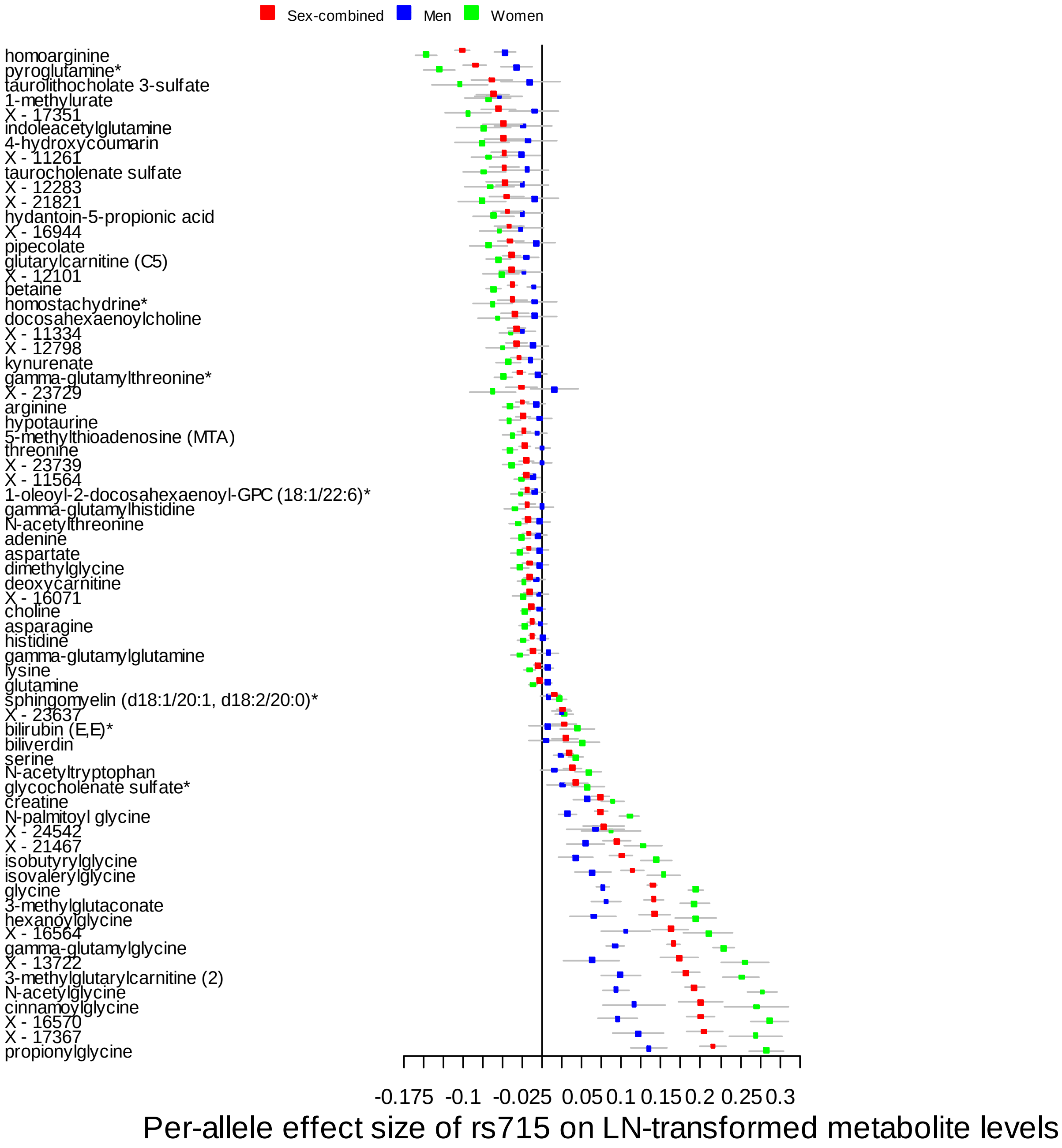

Supplementary Figure 2: Sex-combined and sex-specific per-allele effect sizes of rs715 (*CPS1*) on metabolite levels. Analyses based on 5,706 women and 5,086 men of the EPIC-Norfolk study. Metabolites were included in the plot if they were associated with rs715 at $p<5.6\text{x}10^{-4}$ in men or women, i.e., 69 out of 894 metabolites measured in random sub-cohorts A and B and at least 50% of the total sample size.

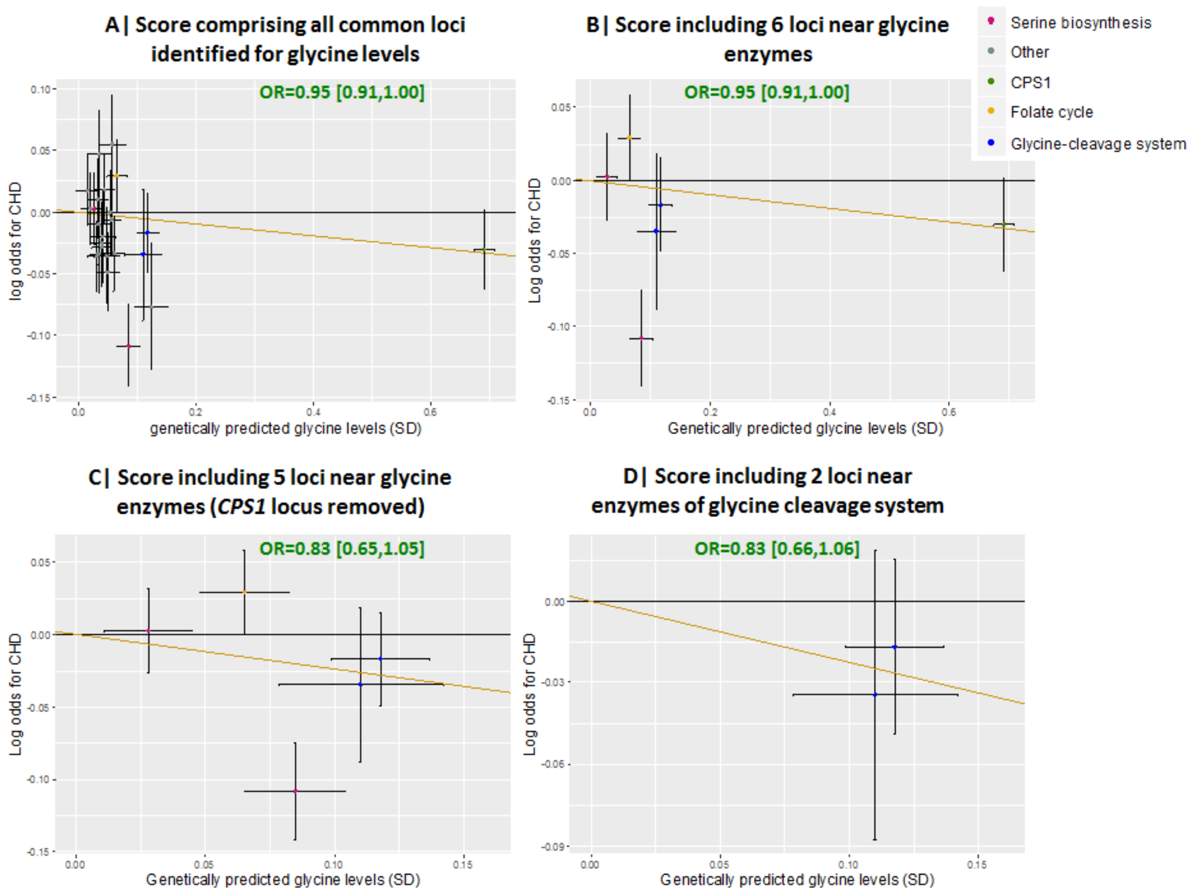

Supplementary Figure 3: Dosage plots of effect sizes of glycine variants on standard deviations of glycine levels versus the log odds for CHD in women: **A|** for the 24 SNP score, **B|** the 6 SNP score, **C|** the 5 SNP score and, **D|** the 2 SNP score. The orange line represents the slope estimated using the weighted median method. Associations with CHD were based on up to 9,853 cases and 202,124 controls from UK Biobank, EPIC-CVD and the GerMIF study.

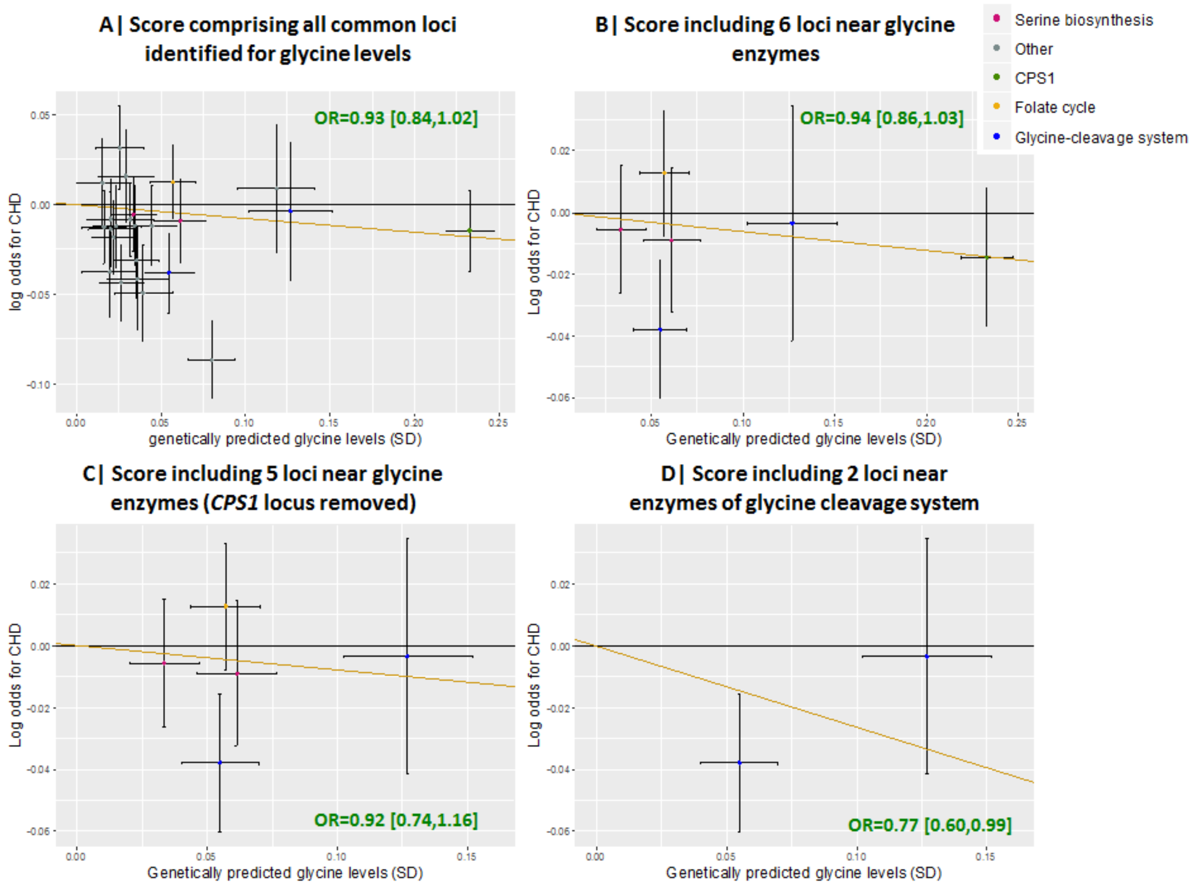

Supplementary Figure 4: Dosage plots of effect sizes of glycine variants on standard deviations of glycine levels versus the log odds for CHD in men: **A|** for the 24 SNP score, **B|** the 6 SNP score, **C|** the 5 SNP score and, **D|** the 2 SNP score. The orange line represents the slope estimated using the weighted median method. Associations with CHD were based on up to 21,944 cases and 194,944 controls from UK Biobank, EPIC-CVD and the GerMIF study.

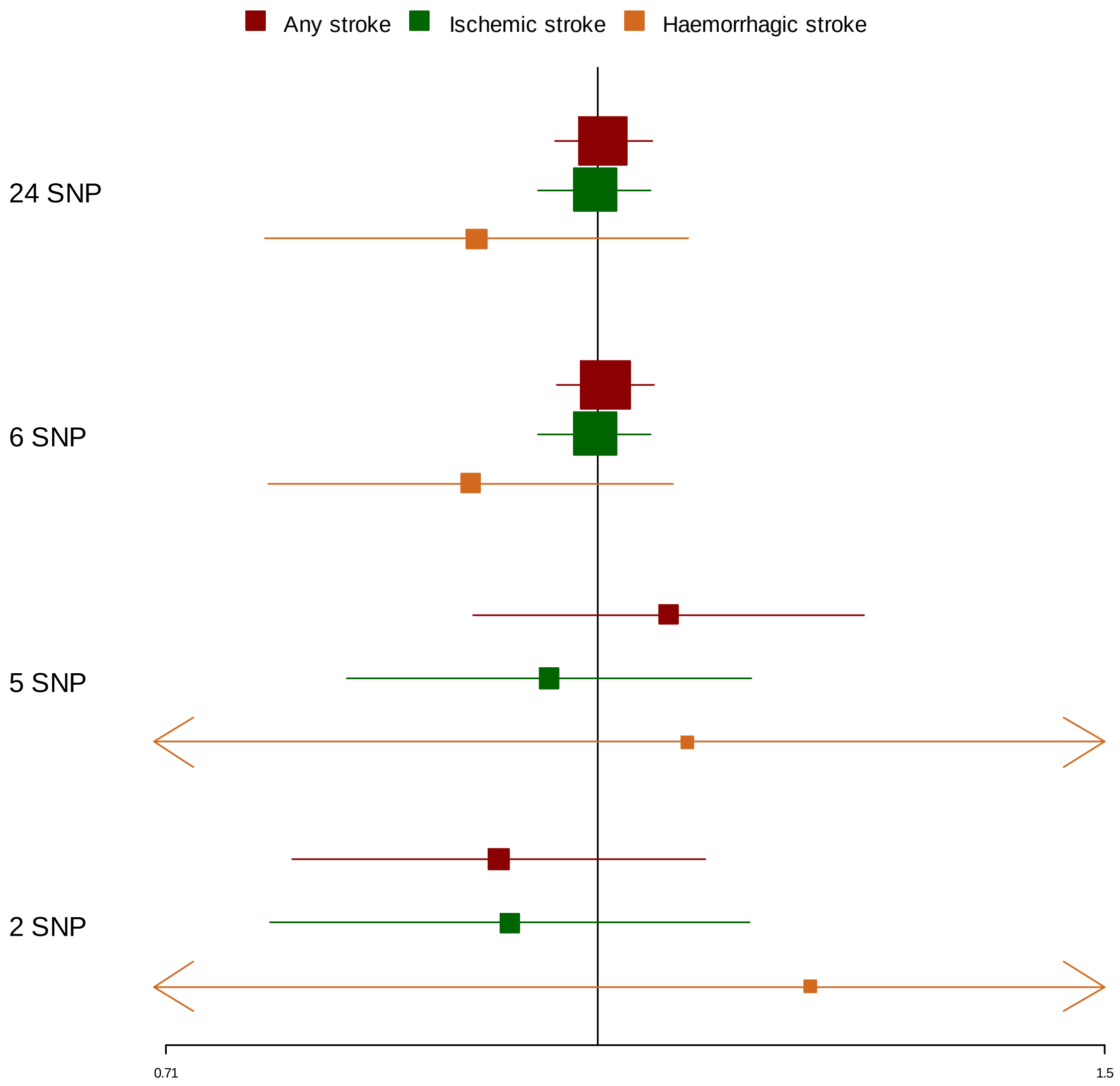

Supplementary Figure 5: **Forest plot of genetically predicted odds ratios on stroke and stroke sub-types per SD of genetically predicted glycine levels.** Results are based on 4 genetic scores for glycine and the weighted median MR method. Analyses were based on summary-level GWAS results from UK Biobank and MEGASTROKE for any and ischemic stroke (Any stroke: up to 48,916 cases and 765,017 non-cases; ischemic stroke: up to 37,771 cases and 764,290 non-cases), and from UK Biobank only for haemorrhagic stroke (1,655 cases and 365,988 non-cases).

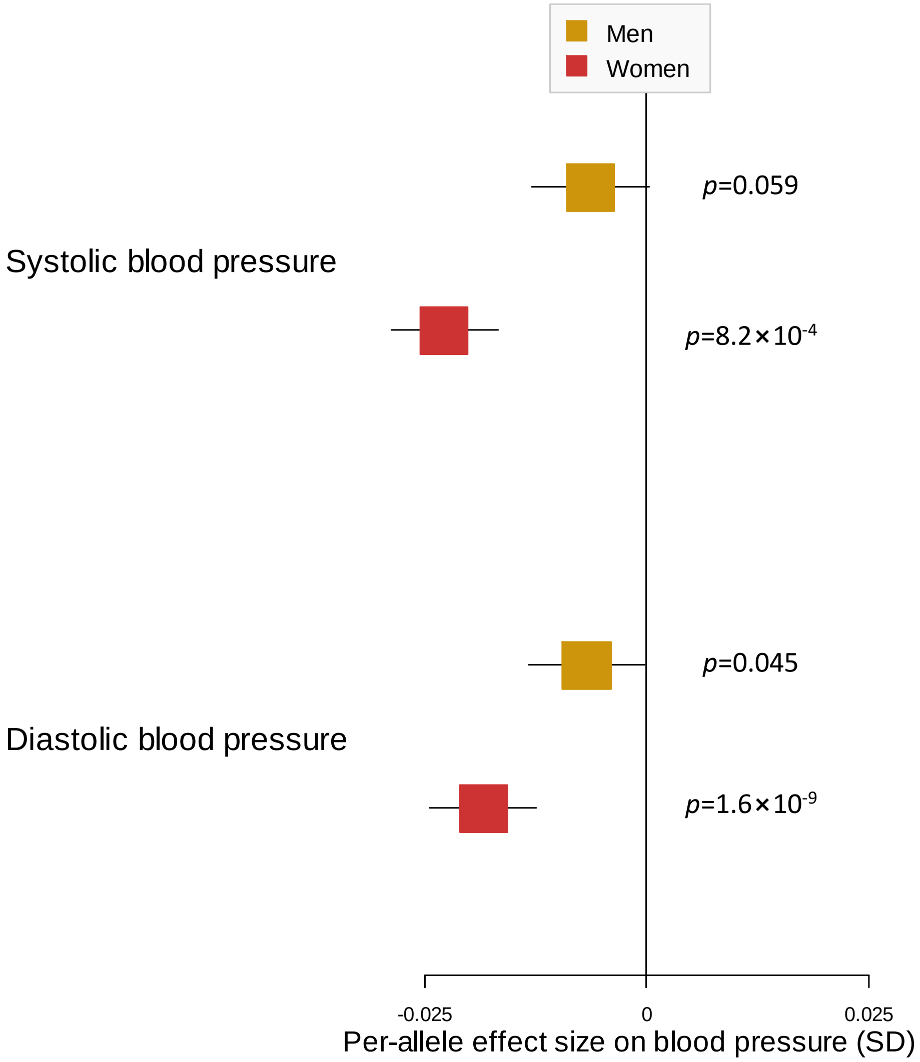

Supplementary Figure 6: **Sex-specific associations of the glycine-increasing allele at rs715 with systolic and diastolic blood pressure.** Genetic associations based on 241,417 female and 203,943 male UK Biobank participants. P-values based on two-tailed t-test.

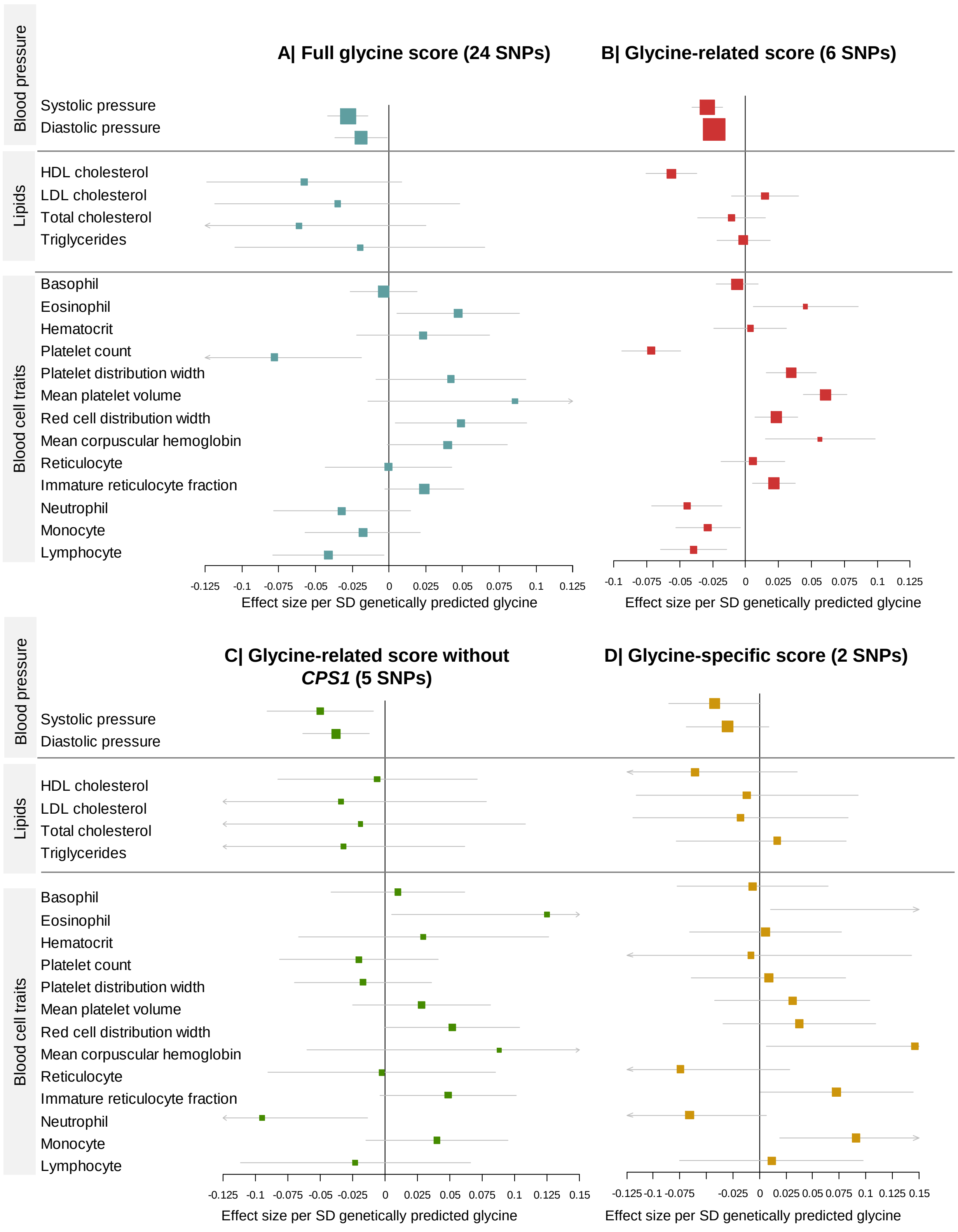

Supplementary Figure 7: Forest plots showing the effect sizes of genetically predicted glycine levels by 4 genetic scores on 19 risk factors of CHD. Genetic associations with blood pressure traits were based on 445,360 UK Biobank participants. Associations with blood lipids were based on look-ups in the summary-level GWAS results from the Global Lipids Genetics Consortium on up to 188,577 participants (Willer *et al*. Nature Genetics 45,1274-1283). Associations with blood cell traits were based on look-ups in the GWAS results on 173,480 participants by Astle *et al.* (Cell 167, 1415-1429).

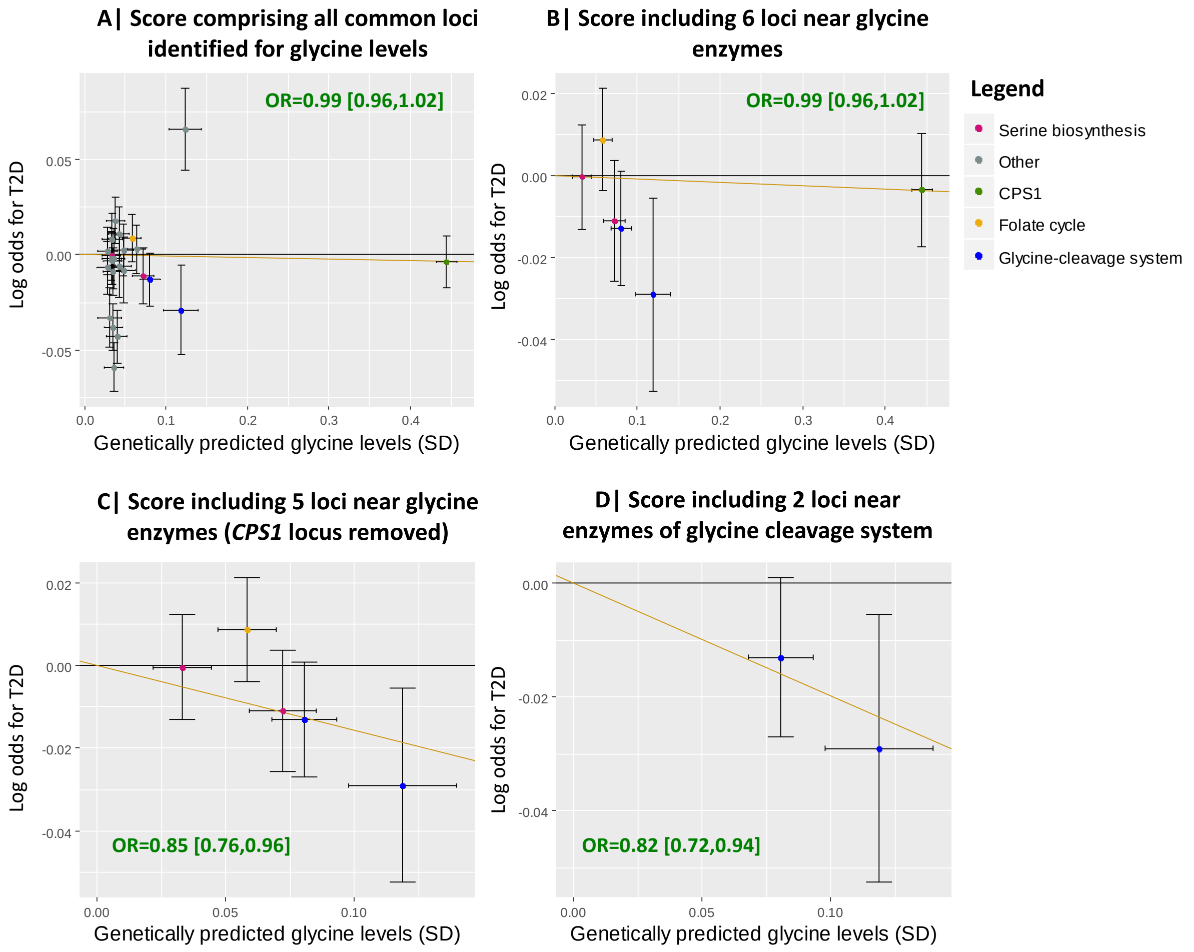

Supplementary Figure 8: Dosage plots of the effect sizes of genetic variants for glycine on standard deviations of glycine levels versus the log odds for type 2 diabetes for sex-combined analyses. A| For the 24 SNP score, **B|** 6 SNP score, **C|** 5 SNP score and, **D|** 2 SNP score. The orange line represents the slope estimated using the weighted median method. Genetic effect sizes on T2D were based on look-ups from the latest GWAS from the DIAGRAM consortium (Mahajan *et al.,* Nature Genetics 50, 1505-1513 (2018)), including 74,124 cases and 824,006 controls. The orange line represents the slope estimated using the weighted median method. SD: standard deviation, T2D: type 2 diabetes.

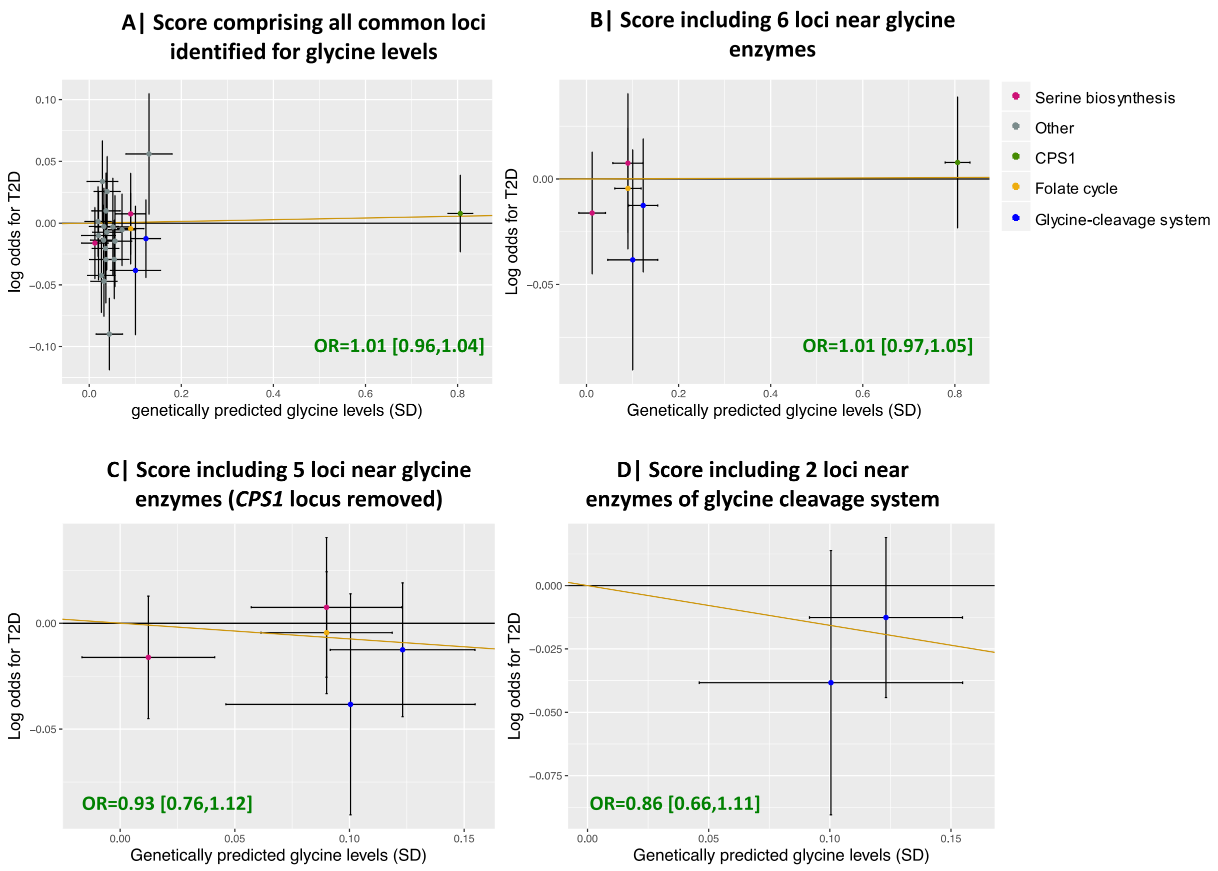

Supplementary Figure 9: Dosage plots of the effect sizes of genetic variants for glycine on standard deviations of glycine levels versus the log odds for type 2 diabetes for women-only analyses. A| For the 24 SNP score, **B|** 6 SNP score, **C|** 5 SNP score and, **D|** 2 SNP score. The orange line represents the slope estimated using the weighted median method. Genetic effect sizes on T2D were based on 12,013 cases and 188,632 controls from InterAct and UK Biobank studies. The orange line represents the slope estimated using the weighted median method. SD: standard deviation, T2D: type 2 diabetes.

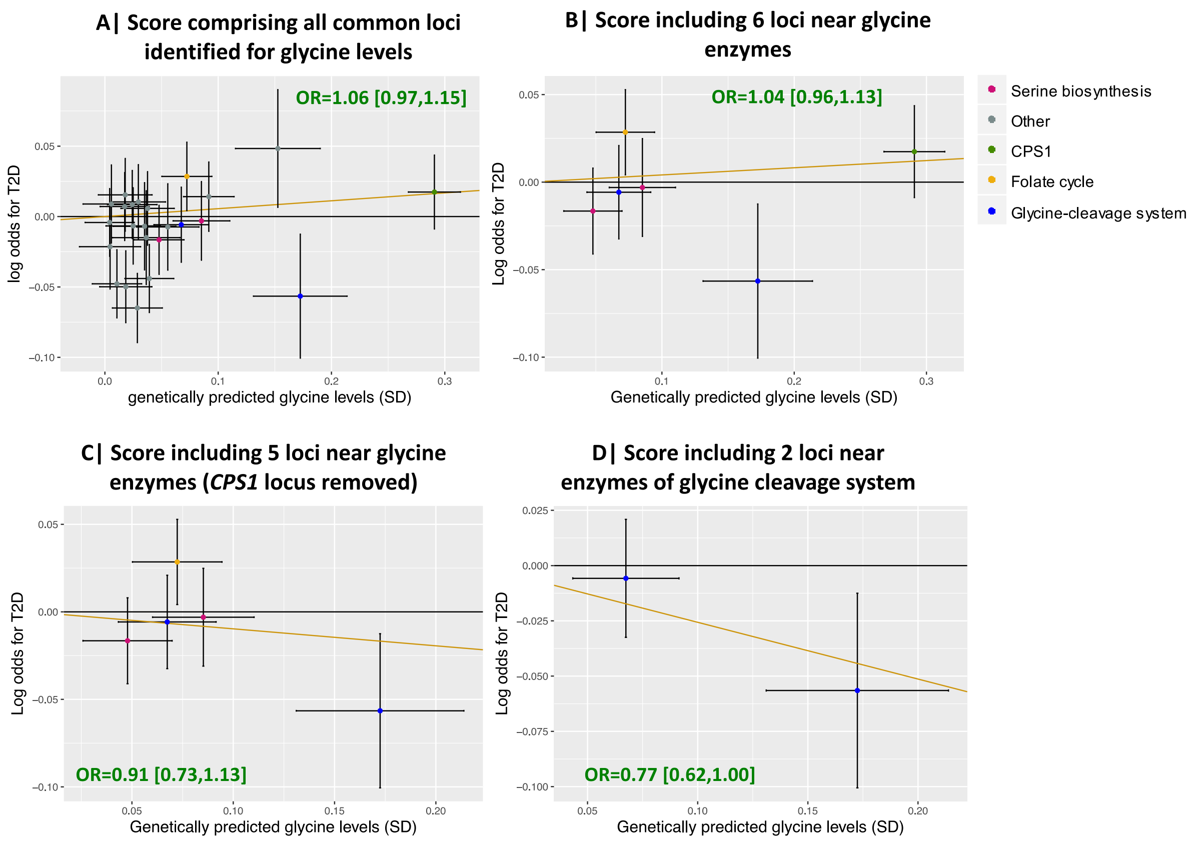

Supplementary Figure 10: Dosage plots of the effect sizes of genetic variants for glycine on standard deviations of glycine levels versus the log odds for type 2 diabetes for men-only analyses. A| For the 24 SNP score, **B|** 6 SNP score**, C|** 5 SNP score and, **D|** 2 SNP score. Genetic effect sizes on T2D were based on 16,914 cases and 153,582 controls from InterAct and UK Biobank studies. The orange line represents the slope estimated using the weighted median method. SD: standard deviation, T2D: type 2 diabetes.

Supplementary Table 1: Overview of samples, phenotype measurement and transformation, genotyping and imputation for the 5 studies included in the p-value and sample size-based meta-analysis of GWAS for glycine levels

Study	Sample size	Metabolomics platform	Sample type	Fasting status	Phenotype transformation	Genotyping array	Imputation reference panel	Number of variants
**Fenland**	(A) 949 + (B) 8,375	Biocrates p180	Plasma	Fasted	1) Natural log transformation, 2) Winsorise at 5 SDs, 3) transformation to Z score	(A) Affymetrix Genome-Wide Human SNP Array 5.0/(B) Affymetrix UK Biobank Axiom Array	(A) 1000G phase 1 / (B) 1000G phase 3	30,850,551
**EPIC-Norfolk**	5,840	Metabolon Discovery HD4	Plasma	Non-fasted	1) Natural log transformation, 2) Winsorise at 5 SDs, 3) transformation to Z score	Affymetrix UK Biobank Array	1000G phase 3	26,328,953
**INTERVAL**	40,509	High-throughput NMR platform	Serum	Non-fasted	1) Natural log transformation, 2) Winsorise at 5 SDs, 3) transformation to Z score	Affymetrix UK Biobank Arrary	UK10K and 1000G phase 3	34,244,352
**Kettunen *et al.***	18,734	High-throughput NMR platform	Serum or plasma	Mostly fasted	Inverse rank-based normal transformation	illumina 610k; Illumina HumanOmniExpress; Illumina HumanCNV37 0; Illumina 318K; Illumina 370K; Affymetrix 250K; Illumina coreexome; Illumina 670k; Affymetrix 6.0; Illumina Human660W; Perlegen-Affymetrix 500K; Illumina 660K; Affymetrix 6.0 907K; Illumina Omni 1M	1000 Genomes Project March 2012	10,577,189
**Shin *et al.* - TwinsUK only**	5,596	Metabolon Discovery HD3	Plasma	Fasted	Log transformation	HumanHap300, HumanHap610Q, 1M-Duo and 1.2MDuo	HapMap2	2,230,893

Supplementary Table 2: Results of Mendelian randomisation analyses of glycine to CHD, sex-specific and combined, and using 4 different methods and 4 different genetic scores for glycine. IVW: inverse variance weighted; WM: weighted median; PWM: penalised weighted median

Full Score	Sex-combined				Women				Men			
	IVW	EGGER	WM	PWM	IVW	EGGER	WM	PWM	IVW	EGGER	WM	PWM
**OR**	0.904	0.977	0.948	0.881	0.928	0.957	0.953	0.954	0.817	0.954	0.926	0.937
**LCI**	0.836	0.897	0.919	0.793	0.848	0.864	0.910	0.912	0.692	0.763	0.842	0.816
**UCI**	0.978	1.064	0.978	0.979	1.014	1.060	0.997	0.998	0.964	1.193	1.019	1.076
p	0.012	0.587	0.001	0.019	0.100	0.401	0.039	0.041	0.017	0.677	0.118	0.360
**CochQ *p***	0.507				0.174				0.092			
**interEGGER**		-0.013				-0.010				0.007		
**EGGER *p***		0.003				0.226				0.057		

6 SNP score	Sex-combined				Women				Men			
	IVW	EGGER	WM	PWM	IVW	EGGER	WM	PWM	IVW	EGGER	WM	PWM
**OR**	0.945	0.967	0.953	0.958	0.942	0.975	0.954	0.954	0.926	0.972	0.941	0.942
**LCI**	0.887	0.885	0.924	0.929	0.826	0.816	0.911	0.913	0.814	0.777	0.860	0.857
**UCI**	1.006	1.056	0.984	0.988	1.075	1.165	0.998	0.998	1.054	1.216	1.029	1.036
p	0.076	0.452	0.003	0.006	0.375	0.779	0.039	0.040	0.245	0.804	0.181	0.218
**CochQ *p***	0.845				0.127				0.800			
**interEGGER**		-0.007				-0.017				-0.007		
**EGGER *p***		0.441				0.540				0.585		

5 SNP score	Sex-combined				Women				Men			
	IVW	EGGER	WM	PWM	IVW	EGGER	WM	PWM	IVW	EGGER	WM	PWM
**OR**	0.836	0.657	0.803	0.767	0.735	0.590	0.827	0.840	0.885	1.043	0.924	0.957
**LCI**	0.699	0.389	0.710	0.677	0.424	0.128	0.649	0.658	0.663	0.432	0.736	0.756
**UCI**	0.999	1.110	0.908	0.870	1.273	2.727	1.054	1.072	1.182	2.518	1.161	1.212
p	0.049	0.116	4.8E-4	3.7E-5	0.272	0.500	0.124	0.162	0.408	0.926	0.498	0.716
**CochQ *p***	0.789				0.104				0.686			
**interEGGER**		0.018				0.019				-0.011		
**EGGER *p***		0.338				0.759				0.695		

2 SNP score	Sex-combined				Women				Men			
	**IVW**	**EGGER**	**WM**	**PWM**	**IVW**	**EGGER**	**WM**	**PWM**	**IVW**	**EGGER**	**WM**	**PWM**
**OR**	0.798	NA	0.798	0.798	0.832	NA	0.832	0.832	0.769	NA	0.769	0.798
**LCI**	0.670	NA	0.693	0.701	0.656	NA	0.655	0.652	0.413	NA	0.595	0.615
**UCI**	0.950	NA	0.919	0.908	1.055	NA	1.056	1.062	1.433	NA	0.993	1.036
p	0.011	NA	0.002	0.001	0.130		0.131	0.139	0.408	NA	0.044	0.091
**CochQ *p***	0.677				0.784				0.269			
**interEGGER**		NA				NA				NA		
**EGGER *p***		NA				NA				NA		

Supplementary Table 3: Results of Mendelian randomisation analyses of glycine to stroke and stroke sub-types, using 4 different methods and 4 different genetic scores for glycine. IVW: inverse variance weighted; WM: weighted median; PWM: penalised weighted median

24 SNP score	Any stroke				Ischemic stroke				Haemorrhagic stroke			
	IVW	EGGER	WM	PWM	IVW	EGGER	WM	PWM	IVW	EGGER	WM	PWM
**OR**	0.997	1.008	1.004	1.004	0.994	0.999	0.997	0.997	0.972	0.883	0.907	0.907
**LCI**	0.950	0.948	0.965	0.966	0.933	0.921	0.953	0.954	0.837	0.733	0.766	0.767
**UCI**	1.046	1.071	1.044	1.045	1.059	1.083	1.043	1.041	1.129	1.064	1.074	1.072
p	0.910	0.802	0.847	0.824	0.863	0.977	0.896	0.892	0.711	0.192	0.258	0.251
**CochQ *p***	0.995				0.899				0.922			
**interEGGER**		-0.002				-0.001				0.017		
**EGGER *p***		0.572				0.858				0.040		

6 SNP score	Any stroke				Ischemic stroke				Haemorrhagic stroke			
	IVW	EGGER	WM	PWM	IVW	EGGER	WM	PWM	IVW	EGGER	WM	PWM
**OR**	1.005	0.984	1.006	1.006	0.995	0.980	0.997	0.997	0.924	0.868	0.903	0.903
**LCI**	0.958	0.923	0.968	0.968	0.946	0.912	0.953	0.954	0.788	0.700	0.768	0.767
**UCI**	1.054	1.050	1.045	1.045	1.046	1.054	1.043	1.043	1.085	1.076	1.061	1.063
p	0.844	0.624	0.776	0.759	0.848	0.587	0.899	0.897	0.335	0.195	0.215	0.220
**CochQ *p***	0.942				0.916				0.878			
**interEGGER**		0.006				0.004				0.019		
**EGGER *p***		0.340				0.539				0.285		

5 SNP score	Any stroke				Ischemic stroke				Haemorrhagic stroke			
	IVW	EGGER	WM	PWM	IVW	EGGER	WM	PWM	IVW	EGGER	WM	PWM
**OR**	1.019	0.686	1.058	1.058	0.981	0.631	0.961	0.961	1.153	1.039	1.074	1.074
**LCI**	0.860	0.485	0.905	0.907	0.820	0.423	0.817	0.808	0.689	0.236	0.574	0.579
**UCI**	1.207	0.968	1.237	1.246	1.173	0.941	1.131	1.143	1.931	4.574	2.008	1.991
p	0.829	0.032	0.480	0.449	0.830	0.024	0.633	0.654	0.587	0.959	0.824	0.822
**CochQ *p***	0.877				0.832				0.818			
**interEGGER**		0.029				0.032				0.008		
**EGGER *p***		0.006				0.001				0.874		

**2 SNP score**	**Any stroke**				**Ischemic stroke**				**Haemorrhagic stroke**			
	**IVW**	**EGGER**	**WM**	**PWM**	**IVW**	**EGGER**	**WM**	**PWM**	**IVW**	**EGGER**	**WM**	**PWM**
**OR**	0.923	NA	0.923	0.923	0.932	NA	0.932	0.932	1.185	NA	1.185	1.185
**LCI**	0.668	NA	0.782	0.785	0.742	NA	0.769	0.767	0.585	NA	0.586	0.574
**UCI**	1.276	NA	1.090	1.086	1.170	NA	1.129	1.133	2.401	NA	2.398	2.448
p	0.629	NA	0.346	0.334	0.543	NA	0.472	0.478	0.637	NA	0.636	0.646
**CochQ *p***	0.494				0.655				0.566			
**interEGGER**		NA				NA				NA		
**EGGER *p***		NA				NA				NA		

Supplementary Table 4: Results of Cox proportional hazards models for the association of glycine levels with coronary heart disease, myocardial infarction and stroke (including stroke subtypes)

**Disease**	**Sex-combined**		**Women**		**Men**	
	**HR [95% CI]**	***p***	**HR [95% CI]**	***p***	**HR [95% CI]**	***p***
Coronary heart disease	0.92 [0.87,0.96]	4.7E-04	0.94 [0.88,1.01]	0.083	0.88 [0.82,0.95]	7.2E-04
Myocardial infarction	0.89 [0.82,0.97]	5.5E-03	0.95 [0.85,1.07]	0.39	0.82 [0.73,0.93]	1.6E-03
Stroke (any)	0.99 [0.93,1.05]	0.70	0.98 [0.91,1.06]	0.68	1.00 [0.90,1.10]	0.92
Haemorrhagic stroke	1.11 [0.96,1.29]	0.15	1.07 [0.89,1.28]	0.49	1.20 [0.94,1.53]	0.13
Ischemic stroke	1.00 [0.92,1.10]	0.95	1.02 [0.91,1.14]	0.74	0.97 [0.84, 1.13]	0.73

Supplementary Table 5: results of inverse variance-weighted Mendelian randomisation analyses for the assessment of the effect of glycine levels on 19 CHD risk factors

CHD risk factors	24 SNP score			6 SNP score			5 SNP score			2 SNP score		
	beta	SE	p-value	beta	SE	p-value	beta	SE	p-value	beta	SE	p-value
**Systolic blood pressure**	-0.028	0.007	1.49E-05	-0.029	0.006	2.56E-06	-0.050	0.021	1.93E-02	-0.042	0.022	5.71E-02
**Diastolic blood pressure**	-0.019	0.009	3.87E-02	-0.024	0.004	4.32E-11	-0.038	0.013	3.09E-03	-0.030	0.020	1.28E-01
**HDL cholesterol**	-0.058	0.034	8.89E-02	-0.056	0.010	7.37E-09	-0.006	0.039	8.76E-01	-0.061	0.049	2.16E-01
**LDL cholesterol**	-0.035	0.043	4.11E-01	0.015	0.013	2.53E-01	-0.034	0.057	5.53E-01	-0.012	0.053	8.23E-01
**Total cholesterol**	-0.061	0.044	1.65E-01	-0.011	0.013	4.18E-01	-0.019	0.065	7.68E-01	-0.018	0.052	7.30E-01
**Triglycerides**	-0.020	0.043	6.48E-01	-0.002	0.010	8.69E-01	-0.032	0.048	4.98E-01	0.017	0.048	7.31E-01
**Basophil**	-0.004	0.012	7.52E-01	-0.006	0.008	4.25E-01	0.010	0.026	7.11E-01	-0.007	0.036	8.56E-01
**Eosinophil**	0.047	0.021	2.75E-02	0.046	0.020	2.52E-02	0.125	0.061	4.12E-02	0.213	0.103	3.92E-02
**Haematocrit**	0.023	0.023	3.14E-01	0.004	0.014	7.96E-01	0.030	0.049	5.49E-01	0.006	0.036	8.76E-01
**Platelet count**	-0.078	0.030	9.94E-03	-0.072	0.011	3.16E-10	-0.020	0.031	5.16E-01	-0.008	0.077	9.17E-01
**Platelet distribution width**	0.042	0.026	1.06E-01	0.035	0.010	2.88E-04	-0.017	0.027	5.25E-01	0.009	0.037	8.15E-01
**Mean platelet volume**	0.086	0.051	9.25E-02	0.061	0.008	5.35E-13	0.028	0.027	3.04E-01	0.031	0.037	4.06E-01
**Red cell distribution width**	0.049	0.023	3.15E-02	0.023	0.008	4.37E-03	0.052	0.027	5.29E-02	0.038	0.037	3.04E-01
**Mean corpuscular haemoglobin**	0.040	0.021	5.63E-02	0.057	0.021	7.78E-03	0.088	0.076	2.45E-01	0.146	0.071	4.00E-02
**Reticulocyte**	-0.001	0.022	9.80E-01	0.006	0.012	6.48E-01	-0.003	0.045	9.52E-01	-0.074	0.052	1.56E-01
**Immature reticulocyte fraction**	0.024	0.014	7.83E-02	0.021	0.008	9.42E-03	0.049	0.027	6.93E-02	0.072	0.037	4.79E-02

**Neutrophil**	-0.032	0.024	1.80E-01	-0.045	0.014	1.03E-03	-0.095	0.042	2.26E-02	-0.066	0.037	7.52E-02
**Monocyte**	-0.018	0.020	3.73E-01	-0.029	0.013	2.24E-02	0.040	0.028	1.52E-01	0.091	0.037	1.35E-02
**Lymphocyte**	-0.041	0.019	3.32E-02	-0.040	0.013	1.87E-03	-0.023	0.045	6.11E-01	0.012	0.044	7.93E-01

Supplementary Table 6: Results of Mendelian randomisation analyses of glycine to T2D, sex-specific and combined, and using 4 different methods and 4 different genetic scores for glycine. IVW: inverse variance weighted; WM: weighted median; PWM: penalised weighted median.

24 SNP score	Sex-combined				Women				Men			
	IVW	EGGER	WM	PWM	IVW	EGGER	WM	PWM	IVW	EGGER	WM	PWM
**OR**	0.975	1.025	0.992	0.992	0.994	1.032	1.007	1.007	1.009	1.135	1.058	1.063
**LCI**	0.892	0.921	0.962	0.961	0.928	0.96	0.969	0.969	0.875	0.954	0.970	0.973
**UCI**	1.064	1.140	1.022	1.024	1.065	1.109	1.046	1.046	1.163	1.349	1.154	1.162
p	0.566	0.651	0.593	0.611	0.871	0.391	0.731	0.729	0.902	0.152	0.203	0.178
**CochQ *p***	0.028				0.139				2.00E-04			
**interEGGER**		-0.009				-0.014				-0.015		
**EGGER *p***		0.121				0.026				0.038		

6 SNP score	Sex-combined				Women				Men			
	IVW	EGGER	WM	PWM	IVW	EGGER	WM	PWM	IVW	EGGER	WM	PWM
**OR**	0.982	0.996	0.992	0.992	1.005	1.025	1.008	1.008	1.022	1.051	1.042	1.049
**LCI**	0.939	0.933	0.961	0.962	0.968	0.978	0.971	0.972	0.894	0.823	0.957	0.963
**UCI**	1.028	1.063	1.023	1.023	1.043	1.074	1.046	1.045	1.169	1.342	1.134	1.142
p	0.566	0.651	0.593	0.611	0.801	0.310	0.683	0.675	0.752	0.689	0.342	0.271
**CochQ *p***	0.955				0.846				0.807			
**interEGGER**		-0.004				-0.011				-0.005		
**EGGER *p***		0.533				0.084				0.776		

5 SNP score	Sex-combined				Women				Men			
	IVW	EGGER	WM	PWM	IVW	EGGER	WM	PWM	IVW	EGGER	WM	PWM
**OR**	0.896	0.703	0.854	0.853	0.919	1.054	0.928	0.928	0.918	0.708	0.908	0.831
**LCI**	0.783	0.499	0.763	0.754	0.778	0.735	0.767	0.763	0.704	0.338	0.730	0.675
**UCI**	1.026	0.991	0.957	0.964	1.085	1.511	1.123	1.130	1.197	1.483	1.129	1.024
p	0.112	0.044	6.514E-03	1.090E-02	0.318	0.775	0.445	0.460	0.527	0.360	0.385	0.082
**CochQ *p***	0.927				0.792				0.724			
**interEGGER**		0.018				-0.014				0.023		
**EGGER *p***		0.140				0.354				0.458		

2 SNP score	Sex-combined	Women	Men

	IVW	EGGER	WM	PWM	IVW	EGGER	WM	PWM	IVW	EGGER	WM	PWM
**OR**	0.821	NA	0.821	0.821	0.919	NA	0.855	0.855	0.774	NA	0.774	0.774
**LCI**	0.721	NA	0.718	0.718	0.778	NA	0.661	0.664	0.623	NA	0.620	0.613
**UCI**	0.935	NA	0.939	0.938	1.085	NA	1.106	1.100	0.960	NA	0.966	0.976
p	2.99E-03	NA	4.00E-03	3.71E-03	0.318	NA	0.233	0.223	0.020	NA	0.024	0.031
**CochQ *p***	0.849				0.657	NA			0.674			
**interEGGER**		NA				NA				NA		
**EGGER *p***		NA				NA				NA		

Supplementary Table 7: Reverse Mendelian randomisation analyses to assess the causality of T2D risk factors on glycine levels. IVW: inverse variance weighted; WM: weighted median; PWM: penalised weighted median.

BMI	IVW	MR-Egger	WM	PWM
**Beta**	-0.041	-0.041	-0.009	-0.007
**SE**	0.030	0.074	0.041	0.040
**pvalue**	0.169	0.581	0.825	0.870
**CochQp**	1.000			
**interEGGER**		-1.34E-05		
**interEGGER p**		0.995		

Fasting insulin	IVW	EGGER	WM	PWM
**beta**	-0.960	-1.179	-0.863	-0.822
**SE**	0.212	1.253	0.211	0.211
P	5.98E-06	3.47E-01	4.29E-05	9.56E-05
**CochQ *p***	0.393			
**interEGGER**		0.003		
**interEGGER p**		0.859		

Early-phase insulin secretion	IVW	EGGER	MW	PMW
**Beta**	0.022	-0.030	-0.011	-0.010
**SE**	0.033	0.087	0.038	0.037
**pvalue**	0.516	0.733	0.762	0.786
**CochQ *p***	0.999			
**interEGGER**		-0.003		
**EGGER *p***		0.524		

Supplementary Table 8: Results of Mendelian randomisation analyses of glycine to 3 site-specific cancers. IVW: inverse variance weighted; WM: weighted median; PWM: penalised weighted median.

24 SNP score	Breast cancer				Ovarian cancer				Prostate cancer			
	IVW	EGGER	WM	PWM	IVW	EGGER	WM	PWM	IVW	EGGER	WM	PWM
OR	1.014	1.016	1.018	1.018	0.989	1.011	1.006	1.007	0.985	1.085	1.045	1.046
LCI	0.987	0.984	0.998	0.998	0.929	0.942	0.966	0.967	0.862	0.900	0.973	0.976
UCI	1.042	1.049	1.038	1.038	1.052	1.085	1.049	1.048	1.127	1.308	1.123	1.120
pvalue	0.300	0.334	0.083	0.083	0.723	0.767	0.759	0.743	0.830	0.390	0.226	0.206
CochQp	0.998				0.591				0.046			
interEGGER		0.000			-0.007					-0.009		
interEGGER *p*		0.862			0.221					0.154		

6 SNP score	Breast cancer				Ovarian cancer				Prostate cancer			
	IVW	EGGER	WM	PWM	IVW	EGGER	WM	PWM	IVW	EGGER	WM	PWM
OR	1.017	1.012	1.017	1.017	0.998	1.020	1.007	1.007	1.008	1.080	1.034	1.051
LCI	0.996	0.983	0.997	0.997	0.926	0.922	0.966	0.968	0.878	0.859	0.959	0.975
UCI	1.039	1.042	1.037	1.037	1.076	1.127	1.050	1.048	1.156	1.359	1.115	1.134
pvalue	0.119	0.409	0.092	0.091	0.964	0.705	0.749	0.720	0.914	0.511	0.386	0.193
CochQp	0.954				0.570				0.614			
interEGGER		0.002				-0.010				-0.010		
interEGGER *p*		0.609				0.501				0.447		

5 SNP score	Breast cancer				Ovarian cancer				Prostate cancer			
	IVW	EGGER	WM	PWM	IVW	EGGER	WM	PWM	IVW	EGGER	WM	PWM
**OR**	0.999	0.820	0.986	0.986	0.845	0.699	0.922	0.962	0.898	0.905	0.842	0.825
**LCI**	0.909	0.676	0.900	0.900	0.624	0.306	0.746	0.778	0.676	0.381	0.674	0.669
**UCI**	1.098	0.995	1.081	1.080	1.144	1.600	1.140	1.191	1.191	2.148	1.052	1.017
**pvalue**	0.984	0.044	0.770	0.768	0.276	0.397	0.453	0.724	0.454	0.820	0.129	0.071
**CochQp**	0.894				0.511				0.531			
**interEGGER**		0.018				0.017				-0.001		
**interEGGER *p***		1.52E-04				0.624				0.985		

2 SNP score	Breast cancer				Ovarian cancer				Prostate cancer			
	IVW	EGGER	WM	PWM	IVW	EGGER	WM	PWM	IVW	EGGER	WM	PWM
**OR**	0.963	NA	0.963	0.963	0.789	NA	0.789	0.911	0.768	NA	0.768	0.768
**LCI**	0.869	NA	0.868	0.869	0.414	NA	0.629	0.715	0.636	NA	0.633	0.633
**UCI**	1.066	NA	1.067	1.066	1.505	NA	0.991	1.162	0.926	NA	0.931	0.932
**pvalue**	0.463	NA	0.466	0.463	0.473	NA	0.042	0.454	0.006	NA	0.007	0.007
**CochQp**	0.798				0.570				0.792			
**interEGGER**		NA				NA				NA		
**interEGGER *p***		NA				NA				NA		

Supplementary Note 1: Acknowledgements

The Fenland Study is supported by the MRC (MC_UU_12015/1). Genetic and metabolomics analyses were funded by the MRC (MC_PC_13046). We are grateful to all the volunteers for their time and help, and to the General Practitioners and practice staff for assistance with recruitment. We thank the Fenland Study Investigators, Fenland Study Co-ordination team and the Epidemiology Field, Data and Laboratory teams. We particularly thank Dr Larissa Richardson and Dr Luke Marney for the acquisition of the metabolomics data.

EPIC-Norfolk has received funding from the Medical Research Council (MR/N003284/1) and Cancer Research United Kingdom (14136). The genetics analyses in EPIC-Norfolk were funded by the Medical Research Council (MC_PC_13048). Additional funding for the metabolomics analysis in EPIC-Norfolk was received from the Medical Research Council (MR/L00002/1) and the Innovative Medicines Initiative Joint Undertaking under EMIF grant agreement number 115372 (European Union's Seventh Framework Programme FP7/2007-2013) and EFPIA companies' in kind contribution. We thank all participants and staff for their contribution to the study.

The EPIC-InterAct study was funded by the EU FP6 Programme (grant number LSHM_CT_2006_037197). We thank staff from the Technical, Field Epidemiology and Data Functional Group Teams of the Medical Research Council Epidemiology Unit in Cambridge, UK, for carrying out sample preparation, DNA provision and quality control, genotyping and data handling work for EPIC-InterAct. We particularly thank Nicola Kerrison (EPIC-InterAct Data Manager).

EPIC-CVD: We thank all participants and staff for their contribution to the study. We thank staff from the EPIC-CVD Coordinating Centre for carrying out sample preparation and data-handling work, particularly Sarah Spackman (EPIC-CVD Data Manager). EPIC-CVD has been supported by the European Union Framework 7 (HEALTH-F2-2012-279233), the European Research Council (268834), the UK Medical Research Council (G0800270 and MR/L003120/1), the British Heart Foundation (SP/09/002 and RG/08/014 and RG13/13/30194), and the UK National Institute of Health Research. The coordination of EPIC is financially supported by the European Commission (DG-SANCO) and the International Agency for Research on Cancer. The funders played no role in the design of the study; the collection, analysis, and interpretation of the data; or the decision to approve publication of the finished manuscript.

INTERVAL: Participants in the INTERVAL randomised controlled trial were recruited with the active collaboration of NHS Blood and Transplant England (www.nhsbt.nhs.uk), which has supported field work and other elements of the trial. DNA extraction and genotyping were co-

funded by the National Institute of Health Research (NIHR), the NIHR BioResource (http://bioresource.nihr.ac.uk/) and the NIHR [Cambridge Biomedical Research Centre at the Cambridge University Hospitals NHS Foundation Trust] [*]. Nightingale NMR assays from the INTERVAL trial were funded by the European Commission Framework Programme 7 (HEALTH-F2-2012-279233). The academic coordinating centre for INTERVAL was supported by core funding from: NIHR Blood and Transplant Research Unit in Donor Health and Genomics (NIHR BTRU-2014-10024), UK Medical Research Council (MR/L003120/1), British Heart Foundation (RG/13/13/30194), and the NIHR [Cambridge Biomedical Research Centre at the Cambridge University Hospitals NHS Foundation Trust]. A complete list of the investigators and contributors to the INTERVAL trial is provided in reference [**]. The academic coordinating centre would like to thank blood donor centre staff and blood donors for participating in the INTERVAL trial. *The views expressed are those of the author(s) and not necessarily those of the NHS, the NIHR or the Department of Health and Social Care. **Di Angelantonio E, Thompson SG, Kaptoge SK, Moore C, Walker M, Armitage J, Ouwehand WH, Roberts DJ, Danesh J, INTERVAL Trial Group. Efficiency and safety of varying the frequency of whole blood donation (INTERVAL): a randomised trial of 45 000 donors. Lancet. 2017 Nov 25;390(10110):2360-2371.

UK Biobank: This research has been conducted using the UK Biobank resource under Application Numbers 12885 (for the genetic analyses related to T2D and CHD) and 20480 (for the genetic analyses related to blood pressure and stroke).

Cancer genetics consortia: We acknowledge the BCAC, OCAC and PRACTICAL consortia for sharing their summary-level GWAS results for cancer. The breast cancer genome-wide association analyses were supported by the Government of Canada through Genome Canada and the Canadian Institutes of Health Research, the 'Ministère de l'Économie, de la Science et de l'Innovation du Québec' through Genome Québec and grant PSR-SIIRI-701, The National Institutes of Health (U19 CA148065, X01HG007492), Cancer Research UK (C1287/A10118, C1287/A16563, C1287/A10710) and The European Union (HEALTH-F2-2009-223175 and H2020 633784 and 634935). All studies and funders are listed in Michailidou et al (2017). The Prostate cancer genome-wide association analyses are supported by the Canadian Institutes of Health Research, European Commission's Seventh Framework Programme grant agreement n° 223175 (HEALTH-F2-2009-223175), Cancer Research UK Grants C5047/A7357, C1287/A10118, C1287/A16563, C5047/A3354, C5047/A10692, C16913/A6135, The National Institute of Health (NIH) Cancer Post-Cancer GWAS initiative grant: No. 1 U19 CA 148537-01 (the GAME-ON initiative), and their other sources of funding as indicated on the consortium website.

L. B. L. W acknowledges funding by the Wellcome Trust (WT083442AIA). C O.-W. is supported by a British Heart Foundation pump-priming grant (RHAG/260) and Homerton College, University of Cambridge. P. S. is supported by a Rutherford Fund Fellowship from the Medical Research Council (MR/S003746/1). S. K. and J. M. M. H. are funded by the National Institute for Health Research [Cambridge Biomedical Research Centre at the Cambridge University Hospitals NHS Foundation Trust]. H. S. is funded by the Deutsche Forschungsgemeinschaft (DFG) as part of the Sonderforschungsbereich CRC 1123 (B2), the Fondation Leducq [CADgenomics: Understanding CAD Genes, 12CVD02], the German Federal Ministry of Education and Research (BMBF) within the framework of ERA-NET on Cardiovascular Disease [Joint Transnational Call 2017, 01KL1802], within the framework of target validation [BlockCAD: 16GW0198K] and within the framework of the e:Med research and funding concept [AbCD-Net: grant 01ZX1706C]. J. L. G. acknowledges funding from the Medical Research Council (MC_UP_A090_1006, MR/P01836X/1 and MR/P011705/1). N. G. F. and F.I. acknowledge funding from Medical Research Council Epidemiology Unit MC_UU_12015/5. N.G.F. and N. J. W. acknowledge funding from the NIHR Biomedical Research Centre Cambridge: Nutrition, Diet, and Lifestyle Research Theme (IS-BRC-1215-20014). S. B. is supported by Sir Henry Dale Fellowship jointly funded by the Wellcome Trust and the Royal Society (204623/Z/16/Z). J. D. is funded by the National Institute for Health Research [Senior Investigator Award]. N. J. W. and C. L. acknowledge funding from the Medical Research Council Epidemiology Unit (MC_UU_12015/1).
