# Supplementary material for: Assessing the causal association of glycine with risk of cardio-metabolic diseases
Source: Nat Commun. 2019 Mar 5;10:1060. doi: 10.1038/s41467-019-08936-1 (PMC6400990; doi:10.1038/s41467-019-08936-1)
Supplement: Supplementary file 3 — Description of Additional Supplementary Files [file 41467_2019_8936_MOESM3_ESM.pdf]

- **Supplementary Data File 1: 27 genetic loci for glycine levels.** This file contains the information on the 27 genetic loci identified for glycine levels based on a p-value-based meta-analysis of GWAS for glycine levels in up to 80,003 participants, including the sex-combined and sex-specific effect sizes and annotations for each of the loci.

- **Supplementary Data File 2: Results of the stepwise selection model to identify secondary genetic signals for glycine levels using GCTA-COJO.**

- **Supplementary Data File 3: Overview of reported associations of the genetic loci for glycine with other traits and metabolites.**

- **Supplementary Data File 4: Associations of 4 genetic scores for glycine and rs715 with 894 metabolites in EPIC-Norfolk.**

- **Supplementary Data File 5: full summary-level results of the p-value-based meta-analysis of 5 GWAS for glycine levels.** This file contains the following columns:
  - MarkerName: chr[chromosome number]:[Base pair position based on GRCh37]:[Alleles in alphabetical order]
  - Chr: chromosome number
  - Pos_GRCh37: base pair position according to GRCh37
  - Effect_allele: Effect allele
  - Other_allele: Other allele
  - Effect_allele_frequency: Meta-analysed effect allele frequency across all studies in which the variant was covered
  - Zscore: Z score showing the direction and strength of association of the variant with glycine levels
  - Pvalue: P-value for the Z score
  - Direction_by_study: String of 5 characters indicating the direction of effect of the effect allele in each of the 5 studies. "?" means that the variant was not covered in the study. The order of the studies is the following:
    1. Fenland
    2. INTERVAL
    3. Kettunen *et al.*
    4. TwinsUK from Shin *et al.*
    5. EPIC-Norfolk
  - SampleSize: Total sample size for the variant
